# β-Polymorph of phenazepam: a powder study

**DOI:** 10.1107/S1600536810037402

**Published:** 2010-09-25

**Authors:** Gleb B. Sergeev, Boris M. Sergeev, Yurii N. Morosov, Vladimir V. Chernyshev

**Affiliations:** a Department of Chemistry, Moscow State University, 119991 Moscow, Russian Federation

## Abstract

The title compound [systematic name: 7-bromo-5-(2-chloro­phen­yl)-1*H*-1,4-benzodiazepin-2(3*H*)-one] (β-polymorph), C_15_H_10_BrClN_2_O, has been obtained *via* cryomodification of the known α-polymorph of phenazepam [Karapetyan *et al.* (1979 ▶). *Bioorg. Khim.* **5**, 1684–1690]. In both polymorphs, the mol­ecules, which differ only in the dihedral angles between the aromatic rings [75.4 (2)° and 86.2 (3)° in the α- and β-polymorphs, respectively], are linked into centrosymmetric dimers *via* N—H⋯O hydrogen bonds. In the crystal structure of the β-polymorph, weak inter­molecular C—H⋯O hydrogen bonds further link these dimers into layers parallel to *bc* plane.

## Related literature

For details of the synthesis *via* cryomodification, see: Sergeev & Komarov (2006 ▶). For the crystal structure of the α-polymorph of phenazepam, see: Karapetyan *et al.* (1979 ▶). For details of the indexing algorithm, see: Werner *et al.* (1985 ▶). The methodology of the refinement (including applied restraints) has been described in detail by Ryabova *et al.* (2005 ▶). For the March–Dollase orientation correction, see: Dollase (1986 ▶) and for the split-type pseudo-Voigt profile, see: Toraya (1986 ▶). 
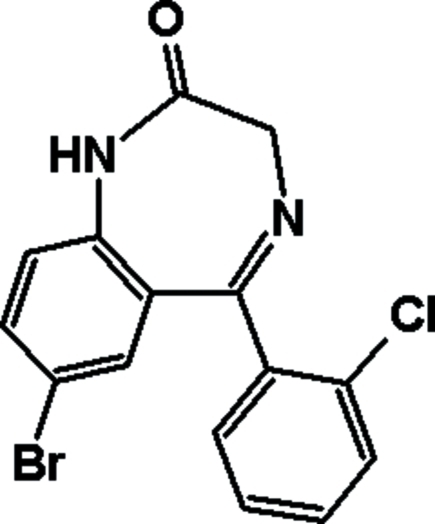


## Experimental

### 

#### Crystal data

C_15_H_10_BrClN_2_O
*M* *_r_* = 349.61
Monoclinic, 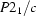

*a* = 14.8006 (19) Å
*b* = 11.6756 (14) Å
*c* = 8.4769 (9) Å
β = 93.679 (17)°
*V* = 1461.8 (3) Å^3^
*Z* = 4
Cu *K*α_1_ radiation, λ = 1.54059 Å
μ = 5.49 mm^−1^
*T* = 295 K
Flat sheet, 15 × 1 mm

#### Data collection

Guinier camera G670 diffractometer
Specimen mounting: thin layer in the specimen holder of the camera
Data collection mode: transmission
Scan method: continuous
2θ_min_ = 5.00°, 2θ_max_ = 80.00°, 2θ_step_ = 0.01°

#### Refinement

*R*_p_ = 0.013
*R*_wp_ = 0.017
*R*_exp_ = 0.012
*R*_Bragg_ = 0.059
χ^2^ = 2.250
7501 data points
128 parameters
64 restraints
H-atom parameters not refined

### 

Data collection: *G670 Imaging Plate Guinier Camera Software* (Huber, 2002 ▶); cell refinement: *MRIA* (Zlokazov & Chernyshev, 1992 ▶); data reduction: *G670 Imaging Plate Guinier Camera Software*; method used to solve structure: simulated annealing (Zhukov *et al.*, 2001 ▶); program(s) used to refine structure: *MRIA*; molecular graphics: *PLATON* (Spek, 2009 ▶); software used to prepare material for publication: *MRIA* and *SHELXL97* (Sheldrick, 2008 ▶).

## Supplementary Material

Crystal structure: contains datablocks I, global. DOI: 10.1107/S1600536810037402/lh5126sup1.cif

Rietveld powder data: contains datablocks I. DOI: 10.1107/S1600536810037402/lh5126Isup2.rtv

Additional supplementary materials:  crystallographic information; 3D view; checkCIF report

## Figures and Tables

**Table 1:** Hydrogen-bond geometry (Å, °)

*D*—H⋯*A*	*D*—H	H⋯*A*	*D*⋯*A*	*D*—H⋯*A*
N8—H8⋯O10^i^	0.86	2.15	2.865 (16)	141
C11—H11*B*⋯O10^ii^	0.97	2.18	3.03 (2)	145

Symmetry codes: (i) 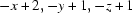; (ii) 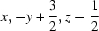.

**Fig. 1.**
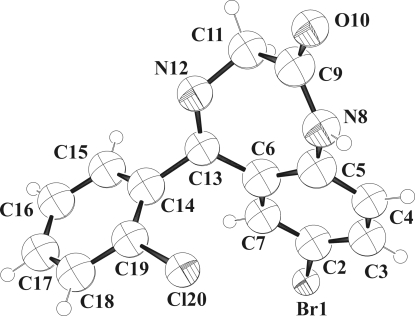
The molecular structure of β-polymorph with the atomic numbering and 50% displacement spheres.

**Fig. 2.**
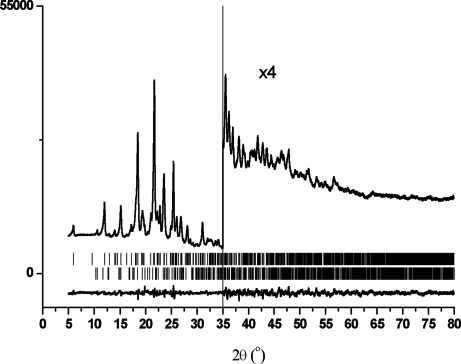
The Rietveld plot, showing the observed and difference profiles for the sample under study. The vertical bars above the difference profile show the reflection positions for α-polymorph (bottom) and β-polymorph (top).

## supplementary crystallographic information

### Comment

Phenazepam is a benzodiazepine drug produced in Russia, which is used in the treatment of neurological disorders such as epilepsy, alcohol withdrawal syndrome and insomnia. The crystal structure of its α-polymorph has been reported by Karapetyan *et al.* (1979). Herewith we present the crystal structure of β-polymorph of phenazepam, which was obtained from the α-polymorph *via* cryomodification, *i.e.* through the preparation of metastable solid-phase from the vapor phase at low temperature (Sergeev & Komarov, 2006).

In β-polymorph (Fig. 1), two six-membered rings form a dihedral angle of 86.2 (3)°, while this dihedral angle is 75.4 (2)° in α-polymorph. In both polymorphs, intermolecular N—H···O hydrogen bonds (Table 1) link the molecules into centrosymmetric dimers. In the crystal structure of β-polymorph (in spite of α-polymorph), the non-classical intermolecular C—H···O hydrogen bonds (Table 1) link further these dimers into layers parallel to *bc* plane.

### Experimental

The title β-polymorph of phenazepam has been obtained *via* cryomodification of α-polymorph of phenazepam. Cryomodification was realized by vapor deposition on a cold surface *in vacuo* at temperatures varying from 77 to 273 K following the known procedure (Sergeev & Komarov, 2006).

### Refinement

During the exposure, the specimen was spun in its plane to improve particle statistics. The triclinic unit-cell dimensions were determined with the indexing program TREOR (Werner *et al.*, 1985), *M*_20_=37, using the first 35 peak positions. A number of weak unindexed lines (*d*-spacings of most significant ones were 8.54, 8.31, 6.90, 5.25 and 5.04 Å) demonstrated that the sample contained a small amount of α-polymorph. The crystal structure of β-polymorph was solved by simulated annealing procedure (Zhukov *et al.*, 2001) and refined following the methodology described in (Ryabova *et al.*, 2005). All non-H atoms were isotropically refined. H atoms were placed in geometrically calculated positions and not refined. The diffraction profiles and the differences between the measured and calculated profiles after the final two-phases Rietveld refinement are shown in Fig. 2. On the results of two-phases Rietveld refinement the ratio of β- and α-polymorphs in the sample was estimated as 1.000 (2) to 0.045 (2), respectively. For the α-polymorph, the atomic coordinates and displacement parameters were fixed to literature values (Karapetyan *et al.*, 1979), so only scale factor and profile parameters were refined.

### Crystal data

**Table:**

C_15_H_10_BrClN_2_O	*F*(000) = 696
*M**_r_* = 349.61	*D*_x_ = 1.589 Mg m^−^^3^
Monoclinic, *P*2_1_/*c*	Cu *K*α_1_ radiation, λ = 1.54059 Å
Hall symbol: -P 2ybc	µ = 5.49 mm^−^^1^
*a* = 14.8006 (19) Å	*T* = 295 K
*b* = 11.6756 (14) Å	Particle morphology: no specific habit
*c* = 8.4769 (9) Å	light grey
β = 93.679 (17)°	flat sheet, 15 × 1 mm
*V* = 1461.8 (3) Å^3^	Specimen preparation: Prepared at 77 K and 6.6 10^-6^ kPa
*Z* = 4	

### Data collection

**Table:**

Guinier camera G670 diffractometer	Data collection mode: transmission
Radiation source: line-focus sealed tube	Scan method: continuous
Curved Germanium (111)	2θ_min_ = 5.00°, 2θ_max_ = 80.00°, 2θ_step_ = 0.01°
Specimen mounting: thin layer in the specimen holder of the camera	

### Refinement

**Table:**

Refinement on *I*_net_	Profile function: split-type pseudo-Voigt (Toraya, 1986)
Least-squares matrix: full with fixed elements per cycle	128 parameters
*R*_p_ = 0.013	64 restraints
*R*_wp_ = 0.017	0 constraints
*R*_exp_ = 0.012	H-atom parameters not refined
*R*_Bragg_ = 0.059	Weighting scheme based on measured s.u.'s
χ^2^ = 2.250	(Δ/σ)_max_ = 0.004
7501 data points	Background function: Chebyshev polynomial up to the 5th order
Excluded region(s): none	Preferred orientation correction: March-Dollase (Dollase, 1986); direction of preferred orientation 001, texture parameter *r* = 0.93(1).

### Special details

**Table:**

Geometry. All e.s.d.'s (except the e.s.d. in the dihedral angle between two l.s. planes) are estimated using the full covariance matrix. The cell e.s.d.'s are taken into account individually in the estimation of e.s.d.'s in distances, angles and torsion angles; correlations between e.s.d.'s in cell parameters are only used when they are defined by crystal symmetry. An approximate (isotropic) treatment of cell e.s.d.'s is used for estimating e.s.d.'s involving l.s. planes.

### Fractional atomic coordinates and isotropic or equivalent isotropic displacement parameters (Å^2^)

**Table:**

	*x*	*y*	*z*	*U*_iso_*/*U*_eq_	
Br1	0.77280 (15)	0.40430 (17)	−0.3314 (2)	0.0470 (11)*	
C2	0.8173 (12)	0.4317 (13)	−0.1182 (18)	0.074 (9)*	
C3	0.8911 (12)	0.3743 (12)	−0.044 (2)	0.075 (8)*	
H3	0.9200	0.3154	−0.0943	0.090*	
C4	0.9202 (11)	0.4080 (13)	0.109 (2)	0.063 (9)*	
H4	0.9684	0.3696	0.1616	0.076*	
C5	0.8784 (12)	0.4986 (15)	0.1866 (18)	0.076 (8)*	
C6	0.8023 (12)	0.5539 (13)	0.110 (2)	0.076 (9)*	
C7	0.7727 (11)	0.5187 (16)	−0.0430 (18)	0.071 (8)*	
H7	0.7228	0.5540	−0.0945	0.085*	
N8	0.9112 (9)	0.5266 (11)	0.3406 (13)	0.064 (6)*	
H8	0.9247	0.4696	0.4020	0.077*	
C9	0.9248 (12)	0.6346 (12)	0.406 (2)	0.072 (8)*	
O10	0.9656 (7)	0.6458 (8)	0.5370 (13)	0.057 (5)*	
C11	0.8837 (11)	0.7351 (13)	0.311 (2)	0.070 (8)*	
H11A	0.8966	0.8060	0.3674	0.084*	
H11B	0.9113	0.7396	0.2101	0.084*	
N12	0.7856 (8)	0.7217 (10)	0.2827 (16)	0.062 (7)*	
C13	0.7513 (11)	0.6468 (13)	0.1858 (19)	0.061 (8)*	
C14	0.6501 (11)	0.6452 (14)	0.1602 (18)	0.071 (9)*	
C15	0.6074 (12)	0.7367 (12)	0.077 (2)	0.074 (8)*	
H15	0.6425	0.7953	0.0384	0.089*	
C16	0.5134 (11)	0.7411 (11)	0.051 (2)	0.075 (8)*	
H16	0.4864	0.8028	−0.0033	0.090*	
C17	0.4600 (11)	0.6530 (13)	0.106 (2)	0.073 (9)*	
H17	0.3976	0.6540	0.0834	0.087*	
C18	0.5002 (12)	0.5633 (14)	0.1936 (17)	0.074 (9)*	
H18	0.4646	0.5067	0.2356	0.089*	
C19	0.5943 (12)	0.5595 (13)	0.2180 (16)	0.065 (8)*	
Cl20	0.6428 (3)	0.4396 (4)	0.3145 (5)	0.054 (2)*	

### Geometric parameters (Å, °)

**Table:**

Br1—C2	1.910 (15)	C11—N12	1.46 (2)
C2—C7	1.39 (2)	C11—H11A	0.9704
C2—C3	1.40 (2)	C11—H11B	0.9697
C3—C4	1.40 (2)	N12—C13	1.28 (2)
C3—H3	0.9297	C13—C14	1.50 (2)
C4—C5	1.41 (2)	C14—C19	1.41 (2)
C4—H4	0.9299	C14—C15	1.41 (2)
C5—N8	1.402 (19)	C15—C16	1.40 (2)
C5—C6	1.42 (2)	C15—H15	0.9299
C6—C7	1.41 (2)	C16—C17	1.39 (2)
C6—C13	1.49 (2)	C16—H16	0.9299
C7—H7	0.9301	C17—C18	1.40 (2)
N8—C9	1.389 (19)	C17—H17	0.9301
N8—H8	0.8600	C18—C19	1.40 (3)
C9—O10	1.23 (2)	C18—H18	0.9303
C9—C11	1.53 (2)	C19—Cl20	1.751 (16)
			
C7—C2—C3	121.6 (14)	C9—C11—H11A	109.4
C7—C2—Br1	114.3 (11)	N12—C11—H11B	109.4
C3—C2—Br1	124.1 (12)	C9—C11—H11B	109.4
C4—C3—C2	118.1 (15)	H11A—C11—H11B	108.0
C4—C3—H3	120.9	C13—N12—C11	121.6 (14)
C2—C3—H3	121.0	N12—C13—C6	125.6 (14)
C3—C4—C5	121.7 (15)	N12—C13—C14	116.8 (14)
C3—C4—H4	119.2	C6—C13—C14	117.3 (14)
C5—C4—H4	119.2	C19—C14—C15	117.4 (15)
N8—C5—C4	118.1 (15)	C19—C14—C13	124.2 (14)
N8—C5—C6	122.4 (15)	C15—C14—C13	118.4 (14)
C4—C5—C6	119.3 (14)	C16—C15—C14	121.2 (15)
C7—C6—C5	118.7 (15)	C16—C15—H15	119.4
C7—C6—C13	118.3 (15)	C14—C15—H15	119.4
C5—C6—C13	123.0 (14)	C17—C16—C15	120.0 (14)
C2—C7—C6	120.6 (15)	C17—C16—H16	120.0
C2—C7—H7	119.7	C15—C16—H16	120.0
C6—C7—H7	119.7	C16—C17—C18	120.0 (15)
C9—N8—C5	128.2 (13)	C16—C17—H17	120.0
C9—N8—H8	115.9	C18—C17—H17	120.0
C5—N8—H8	115.9	C19—C18—C17	119.4 (15)
O10—C9—N8	120.5 (13)	C19—C18—H18	120.3
O10—C9—C11	123.4 (13)	C17—C18—H18	120.3
N8—C9—C11	116.1 (14)	C18—C19—C14	121.9 (14)
N12—C11—C9	111.1 (13)	C18—C19—Cl20	118.0 (12)
N12—C11—H11A	109.4	C14—C19—Cl20	120.0 (13)

### Hydrogen-bond geometry (Å, °)

**Table:**

*D*—H···*A*	*D*—H	H···*A*	*D*···*A*	*D*—H···*A*
N8—H8···O10^i^	0.86	2.15	2.865 (16)	141
C11—H11B···O10^ii^	0.97	2.18	3.03 (2)	145

Symmetry codes: (i) −*x*+2, −*y*+1, −*z*+1; (ii) *x*, −*y*+3/2, *z*−1/2.
